# KAT2A-mediated succinylation modification of notch1 promotes the proliferation and differentiation of dental pulp stem cells by activating notch pathway

**DOI:** 10.1186/s12903-024-03951-1

**Published:** 2024-03-31

**Authors:** Longwei Ye, Zeqin Yu, Lin He, Jie Yuan, Xiaodan Zhang, Lei Li, Xin Huang, Yanyan Ma, Lei Zhang

**Affiliations:** 1 Department of Oral Health and Prevention, The First Affiliated Hospital of Harbin Medical University. Harbin Medical University, School of Stomatology, No.143 Yiman Street, Nangang District, Harbin City, 150001 Heilongjiang Province China; 2 Department of Stomatology, Heilongjiang Province Hospital, Harbin City, 150081 Heilongjiang Province China; 3 Pharmaceutical Department, The First Affiliated Hospital of Harbin Medical University, Harbin City, 150001 Heilongjiang Province China

**Keywords:** Dental pulp stem cells, Osteogenic differentiation, Succinylation, KAT2A, Notch1

## Abstract

**Background:**

Dental pulp stem cells (DPSCs) are a kind of undifferentiated dental mesenchymal stem cells with strong self-renewal ability and multi-differentiation potential. This study aimed to investigate the regulatory functions of succinylation modification in DPSCs.

**Methods:**

DPSCs were isolated from the dental pulp collected from healthy subjects, and then stem cell surface markers were identified using flow cytometry. The osteogenic differentiation ability of DPSCs was verified by alkaline phosphatase (ALP) and alizarin red staining methods, while adipogenic differentiation was detected by oil red O staining. Meanwhile, the mRNA of two desuccinylases (SIRT5 and SIRT7) and three succinylases (KAT2A, KAT3B, and CPT1A) in DPSCs before and after mineralization induction were detected using quantitative real-time PCR. The cell cycle was measured by flow cytometry, and the expression of bone-specific genes, including COL1a1 and Runx2 were evaluated by western blotting and were combined for the proliferation and differentiation of DPSCs. Co-immunoprecipitation (co-IP) and immunofluorescence were combined to verify the binding relationship between proteins.

**Results:**

The specific markers of mesenchymal stem cells were highly expressed in DPSCs, while the osteogenic differentiation ability of isolated DPSCs was confirmed via ALP and alizarin red staining. Similarly, the oil red O staining also verified the adipogenic differentiation ability of DPSCs. The levels of KAT2A were found to be significantly upregulated in mineralization induction, which significantly decreased the ratio of G0/G1 phase and increased S phase cells; converse results regarding cell cycle distribution were obtained when KAT2A was inhibited. Moreover, overexpression of KAT2A promoted the differentiation of DPSCs, while its inhibition exerted the opposite effect. The elevated KAT2A was found to activate the Notch1 signaling pathway, which succinylated Notch1 at the K2177 site to increase their corresponding protein levels in DPSCs. The co-IP results showed that KAT2A and Notch1 were endogenously bound to each other, while inhibition of Notch1 reversed the effects of KAT2A overexpression on the DPSCs proliferation and differentiation.

**Conclusion:**

KAT2A interacted directly with Notch1, succinylating the Notch1 at the K2177 site to increase their corresponding protein levels in DPSCs. Similarly, KAT2A-mediated succinylation modification of Notch1 promotes the DPSCs proliferation and differentiation, suggesting that targeting KAT2A and Notch1 may contribute to tooth regeneration.

**Supplementary Information:**

The online version contains supplementary material available at 10.1186/s12903-024-03951-1.

## Introduction

Dental pulp stem cells (DPSCs) are adult stem cells isolated from dental pulp tissue, which are abundant and can easily be isolated [1]. A study compared the proliferation, cloning, and mineralization properties of DPSCs and bone marrow stem cells and revealed that DPSCs exhibited significant proliferation, cloning, and mineralization potential [2], making DPSCs a potential and ideal cell source for bone tissue engineering.

The proliferation and differentiation characteristics of stem cells are regulated by various factors, such as transcription factor regulation, variable splicing of RNA, and post-translational modification (PTM) of proteins [3–5], where PTM is central in regulating self-renewal, proliferation, and differentiation of stem cells [6]. Succinylation is a novel multienzyme-regulated PTM, and changes in succinylation levels in diverse proteins may compromise multiple metabolism pathways and regulate osteogenic differentiation [7]. Accumulating evidence suggested that succinylation participates in the regulation of stem cells. For instance, induction of CDC42 succinylation inhibits the proliferation of neural stem cells to aggravate cerebral ischemia/reperfusion injury [8]. Similarly, elevated succinylation mediated by SIRT5 knockdown inhibited the proliferation of adipose-derived mesenchymal stem cells [9]. Hence, further investigation on how succinylation modulates the proliferation and differentiation of DPSCs is needed.

In this project, it was speculated that the proliferation and differentiation of DPSCs may be regulated by succinylation modification. Thus, potential desuccinylases and succinylases that are aberrantly expressed in DPSCs under the differentiation induction were screened, and their regulatory functions on the cell cycle and calcium mineralization formation of DPSCs were investigated. In current work, KAT2A which can enhance the transcriptional activity of downstream genes by regulating protein succinylation [10, 11] was found to be highly expressed in the DPSCs after induction of mineralization, and the high expression of KAT2A enhanced the proliferative and differentiation capacities. Furthermore, the underlying signaling pathways regulated by KAT2A mediated succinylation were also identified.

## Methods and materials

### Isolation and culture of DPSCs

The samples were collected at the oral and maxillofacial surgery department of The First Affiliated Hospital of Harbin Medical University, following the patient’s written consent, in accordance with the Declaration of the Helsinki World Medical Association, after ethical approval from the institutional Ethics Committee. Briefly, the extracted third molar teeth of healthy adults (18 to 25 years) or the premolar teeth removed during orthodontic treatment of healthy teenagers (13 to 19 years) were collected and immersed in α-MEM containing 5% penicillin and streptomycin (Hyclone, USA). The blood stains were thoroughly washed using phosphate-buffered saline (PBS) and subjected to fresh pulp extraction following teeth splitting. The 1/3rd pulp of the root tip was removed, and the white fibrous connective tissue obtained after washing with PBS was carefully collected. The collected pulp tissue (1 mm^3^) was mixed with 3 mg/mL collagenase I (Gibco, USA) and 4 mg/mL Dispase II (Gibco, USA) and allowed to be fully digested at 37 ℃ for 60 min. It was proceeded by neutralizing collagenase activity using α-MEM, 20% fetal bovine serum (FBS, BI, Israel), and 500 U/mL penicillin/streptomycin solution. The mixture was centrifuged at 1000 rpm for 5 min, and the supernatant was removed. The digested pulp tissue was resuspended in α-MEM containing 20% FBS and 500 U/mL penicillin/streptomycin solution and cultured at 37 ℃ and 5% CO_2_ for 3 to 5 days. The culture medium was changed every three days until the cells were 80% confluent.

### Identification of DPSCs

Surface markers of DPSCs were detected by flow cytometry. The DPSCs were digested with 0.25% pancreatic enzyme (Beyotime, China) and centrifuged (1000 rpm, 5 min). After the supernant was discarded, the DPSCS were added to PBS (Solarbio, China) for re-suspension. Then the density of DPSCs was adjusted to about 1 × 10^6^ cells/mL. According to the antibody instructions (BioLegend, USA), cell surface molecule-related antibodies, including CD90, CD105, CD34, and CD45, were added, and the other tube was used as a blank control. All tubes were fully incubated at room temperature for 30 min. Centrifugation was performed at 1000 rpm for 5 min, after the supernatant was poured out, the precipitate was fully rinsed with PBS, and then re-suspended with 500 µL of PBS, and detected by flow cytometry (BD Biosciences, USA).

### Osteogenic differentiation

As cells reached 80–90% confluence, DPSCs were seeded into 6-well plates at a density of 1 × 10^5^ cells/well. The complete culture medium was replaced with the OriCell osteogenic induction differentiation medium (Cyagen, China) to continue the culture. The medium was changed every 3 days and mineralization induction was terminated after 21 days. On the 7th day, the supernatant was discarded, and the DPSCs were fixed with 4% paraformaldehyde (Lianke Bio, China) for 15 min and stained with alkaline phosphatase (ALP, Sigma-Aldrich, USA). Mineral deposits in DPSCs were stained with alizarin red to evaluate odontoblastic differentiation. On the 14th day, the DPSCs were fixed with 4% paraformaldehyde and stained with 0.2% alizarin red (Sigma-Aldrich, USA) for 20 min. The ALP-positive area and alizarin red-positive area were analyzed under a stereoscopic microscope (Zeiss, Germany).

### Adipogenic differentiation

As cells reached 80–90% confluence, DPSCs were seeded into 6-well plates at a density of 1 × 10^5^ cells/well. Lipogenesis induction solution A (1 mL) was added into the DPSCs, and after 3 days induction, liquid B was added for 24 h. The DPSCs were alternately induced 3 to 5 times according to the instructions (OriCell adipogenic induction differentiation medium, Cyagen, China), and observed under the microscope (Leica, Germany). After the formation of lipid droplets, the DPSCs were cultured with liquid B for 1 week, and the liquid was changed regularly until the operation was completed when the lipid droplets were large and round. Lipid droplets were then stained with Oil Red O stain kit (Solarbio, China) according to the recommended protocol, and the formation of lipid droplets was observed under inverted microscope and the images were collected.

### Cell transfection

The cDNA encoding KAT2A was amplified from DPSCs and sequenced, and then subcloned into the pcDNA3 vector (Invitrogen, USA), subsequently named oe-KAT2A. A Quik Change Site-Directed Mutagenesis Kit (Stratagene, USA) was used for point mutations. shRNAs were designed as follows: sh-KAT2A (5′-CGTGCTGTCACCTCGAATGA-3′) and sh-Notch1 (5′-GGGAGAAACCCACACTGTTTC-3′). shRNAs was cloned into lentiviral expression vector pLL3.7. DPSCs were seeded in 6 cm plates, and reached about 50% confluence before transfection, which was performed according to the manufacturer’s recommendations. After DPSCs were infected using polybrene (Sigma-Aldrich, USA) for 24 h, 8 µg/mL Puromycin (Sigma) was added to select the positive transfected cells for 1 week at 37 °C, 5% CO_2_. Stably infected cells were chosen and used for further experiments.

### RNA extraction and quantitative real-time PCR analysis

DPSCs were mixed with TRIzol reagent (Invitrogen, USA) to extract the total RNA. cDNA was made using High Capacity cDNA Reverse Transcription Kit (Applied Biosystems, USA). Quantitative real-time PCR was carried out by using SYBR Green PCR Master Mix (Takara, Japan). The StepOnePlus Real‐Time PCR System (Applied Biosystems, USA) was used to detect the transcript levels of the genes. The primer sequences are listed in Table 1.

**Table 1:** Primer sequences

Primer	Forward (5’→3’)	Reverse (5’→3’)
CPT1A	TCCAGTTGGCTTATCGTGGTG	TCCAGAGTCCGATTGATTTTTGC
KAT2A	GCAAGGCCAATGAAACCTGTA	TCCAAGTGGGATACGTGGTCA
KAT3B	AGCCAAGCGGCCTAAACTC	TCACCACCATTGGTTAGTCCC
SIRT5	GCCATAGCCGAGTGTGAGAC	CAACTCCACAAGAGGTACATCG
SIRT7	GACCTGGTAACGGAGCTGC	CGACCAAGTATTTGGCGTTCC
Notch1	GAGGCGTGGCAGACTATGC	CTTGTACTCCGTCAGCGTGA

### Cell cycle analysis

DPSCs were washed with PBS and then fixed in 70% ethanol at 4 °C overnight. Then DPSCs were stained with 100 mg/mL propidium iodide (Sigma-Aldrich, USA) and a 10 µg/mL RNAse (Takara, Japan) cocktail at 4 °C for 30 min. The flow cytometry was carried out to analysis cell cycle.

### Western blotting and co-immunoprecipitation (co-IP)

Protein was extracted from DPSCs cells by RIPA reagent (Thermo Fisher Scientific, USA). The total protein concentration was determined with a BCA Protein Assay Kit (Beyotime, China). Afterwards, protein (20 µg) was separated by 10% SDS-PAGE and electrotransferred to PVDF membrane (Merck, China). The membranes were blocked with 5% skim milk for 1 h and incubated with primary antibodies at 4℃ overnight. Subsequently, horseradish peroxidase (HRP)-conjugated secondary antibodies were used to probe the membrane for 1 h. The proteins were visualized by ImageQuant LAS-4000 mini (GE Healthcare, USA), and the relative protein levels were measured by ImageJ software. co-IP was performed to verify the endogenous interaction between KAT2A and Notch1 protein. The anti-KAT2A, anti-Notch1, and normal IgG were added to 1 mg cell lysates precleared with Protein G Agarose beads and then incubated at 4 °C overnight. the co-IP complexes were harvested and analyzed by western blot analysis. The information of all antibodies used in this study are listed in Table 2.

**Table 2:** Information of antibodies

Protein	Product code	Manufacturer
CD105	ab307399	Abcam
CD34	ab245689	Abcam
CD45	ab243869	Abcam
COL1a1	ab138492	Abcam
Runx2	ab220117	Abcam
Notch1	ab52627	Abcam
Hes1	ab108937	Abcam
Hey1	ab154077	Abcam
p-AMPK	2535 S	CST
AMPK	66536-1-Ig	Proteintech
p-mTOR	ab109268	Abcam
mTOR	ab32028	Abcam
Wnt5a	55184-1-AP	Proteintech
β-catenin	51067-2-AP	Proteintech
succinyllysine polyclonal antibody	PTM-401	PTM Biolabs

### Immunofluorescence co-localization

The DPSCs (1 × 10^5^/mL) were seeded into a 35 mm cell imaging dish with a glass bottom. The cells were fixed in 4% paraformaldehyde for 15 min and permeabilized with phosphate-buffered saline containing 0.3% TritonX-100 for 15 min. Cells were blocked with 5% FBS in PBS for 1 h, then incubated with anti-KAT2A and anti-Notch1 antibody at 4℃ overnight, followed by an Alexa Fluor® 647 conjugate goat anti-Rabbit IgG secondary antibody for 2 h. Finally, the DPSCs were mounted with DAPI and fluorescence images were acquired with a light microscope (Leica DM 2500, Germany) at 200× magnifications.

### Statistical analysis

All experiments were performed with at least three times. The error bars indicated ± standard deviation (SD). All data were in a normal distribution, and variance was similar between the groups that are being statistically compared. Statistical analyses were analyzed in GraphPad Prism 7. Statistical significance was determined by using unpaired Student t test for two groups or one-way ANOVA when there are more than two groups. *p* < 0.05 was considered significant.

## Results

### The identification and the differentiation of DPSCs

**Fig. 1:**
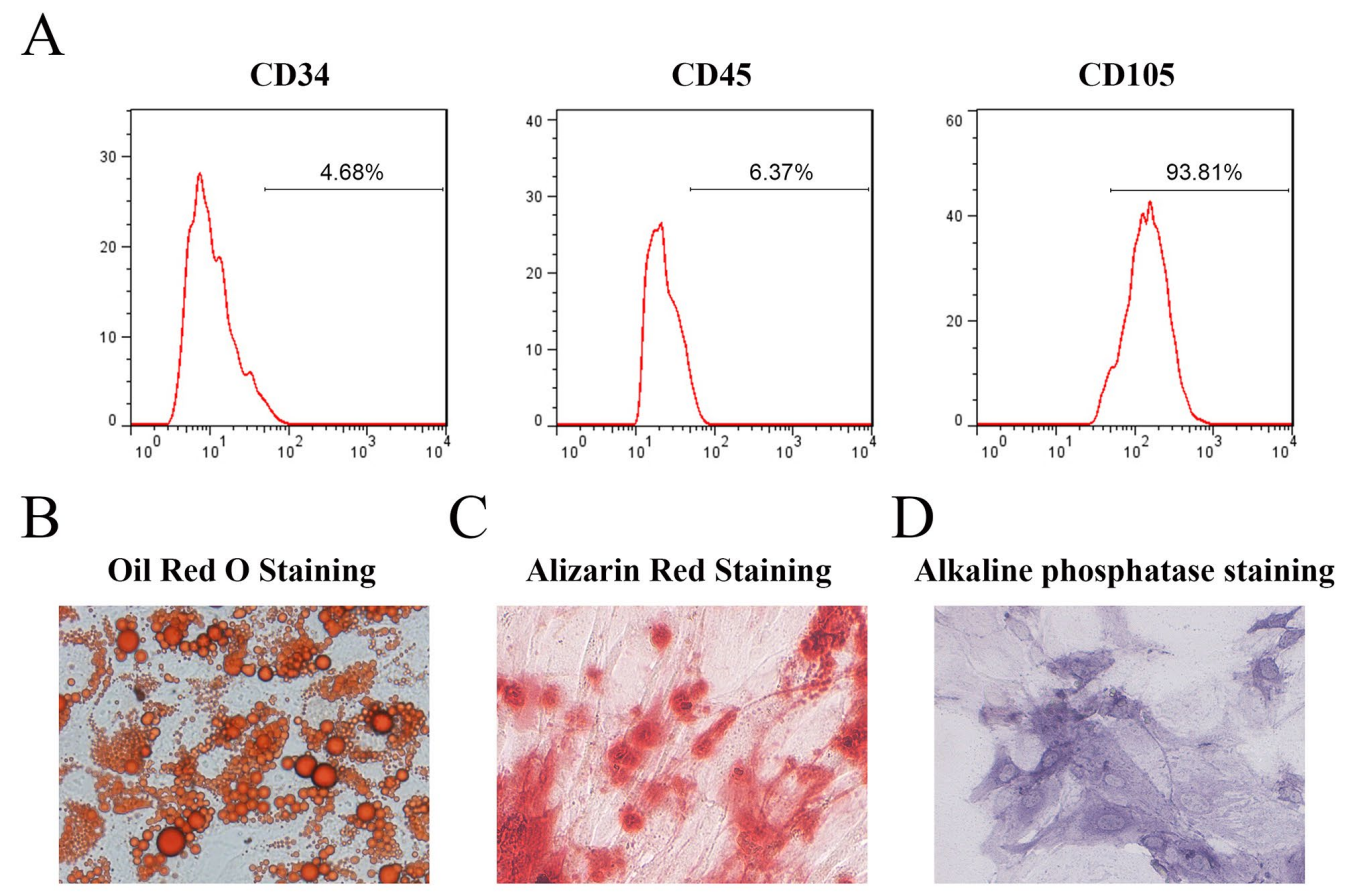
Characterizations and regenerative capacity of DPSCs. **(A)** Mesenchymal stem cell antigen (CD105) and hematopoietic cell antigen (CD34 and CD45) expressed in DPSCs were detected by flow cytometry. **(B)** Accumulation of lipid vacuoles was detected by oil O staining. **(C)** Calcium mineralization formation observed by alizarin red staining at day 14 in DPSCs. **(D)** Alkaline phosphatase (ALP) straining of DPSCs after seven days of osteogenic induction

The DPSCs isolated from collected clinical samples were identified by detecting the surface-specific markers using flow cytometry, and results showed that CD105, a specific marker for mesenchymal stem cells, was highly expressed in DPSCs, while almost no expression was observed for CD34 and CD45, which are the hematopoietic stem cell surface markers (Fig. 1A). The differentiation potential of isolated DPSCs was detected via oil red O staining to induce lipid formation for 28 days, and viewing cells under microscope revealed formation of red lipid droplets, proving the adipogenic differentiation ability of DPSCs (Fig. 1B). Similarly, alizarin red staining results at 14th day demonstrated red color in the mineralized induction group, which was achromatous in the control group, cementing the osteogenic differentiation ability of isolated DPSCs (Fig. 1C). Meanwhile, ALP staining was performed on DPSCs 7 days after mineralization in the osteogenic induction medium, and results showed that the color of DPSCS in the mineralized induction group was deep blue compared with that in the control group (Fig. 1D).

### Elevated KAT2A promotes the proliferation and differentiation of DPSCs

**Fig. 2:**
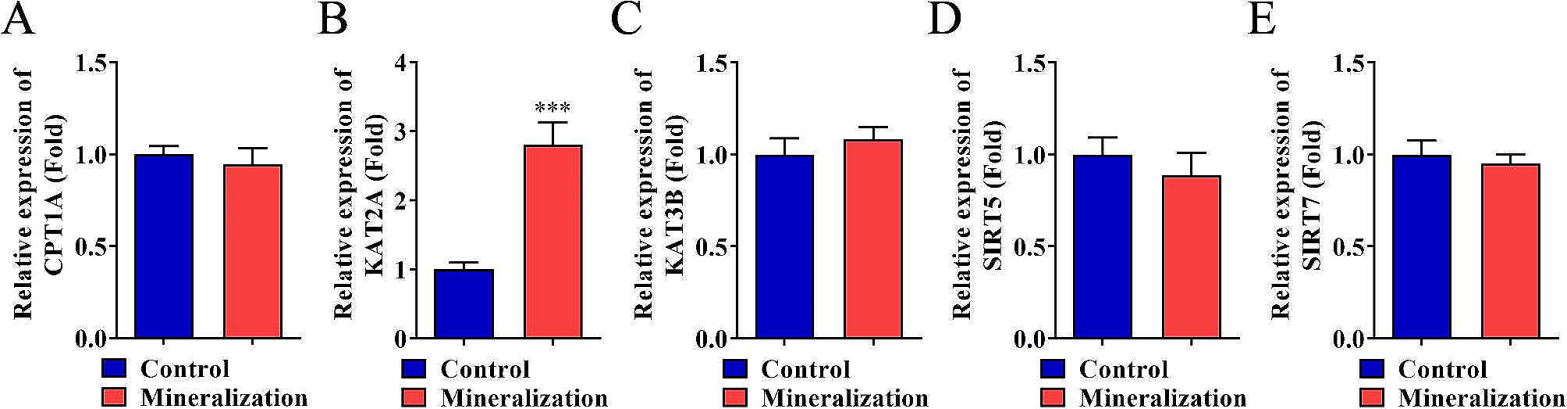
Expression of desuccinylase and succinylase in DPSCs. **A–E.** PCR analysis was performed to evaluate the mRNA levels of two desuccinylases (SIRT5 and SIRT7) and three succinylases (KAT2A, KAT3B, and CPT1A) in DPSCs before and after mineralized induction. ***p < 0.001

**Fig. 3:**
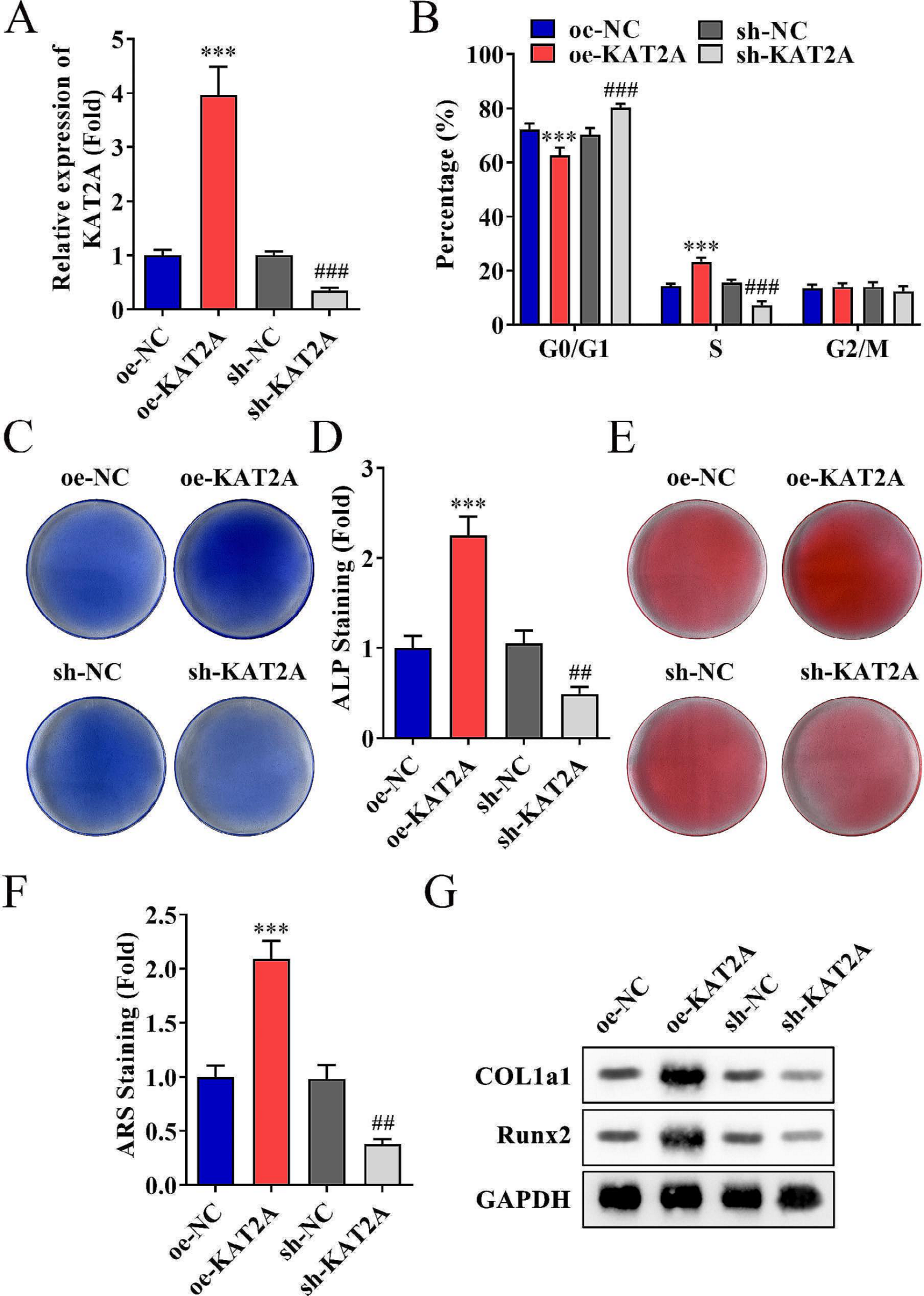
Regulatory effects of KAT2A on proliferation and differentiation of DPSCs. **(A)** PCR analysis was performed to evaluate the mRNA levels of KAT2A in DPSCs after transfection. **(B)** Flow cytometry detected the DPSC cell cycle phase distribution after transfection. **C–D.** Alkaline phosphatase (ALP) straining of DPSCs after seven days of osteogenic induction. **E–F.** Calcium mineralization formation was observed by alizarin red staining at day 14 in DPSCs. **G.** Protein expression of osteogenic markers COL1a1 and RUNX2 analyzed by western blotting. ***p < 0.001 (vs. oe-NC group); ##*p* < 0.01, ###*p* < 0.001 (vs. sh-NC group)

The mRNA levels of two desuccinylases (SIRT5 and SIRT7) and three succinylases (KAT2A, KAT3B, and CPT1A) in DPSCs before and after mineralized induction were measured, and results showed that the KAT2A levels were significantly upregulated in the mineralization group compared to the control group. In contrast, the difference between the two groups regarding CPT1A, KAT3B, SIRT5, and SIRT7 levels was insignificant (Fig. 2A–E, *p* < 0.001). The effect of KAT2A on regulating the proliferation and differentiation of DPSCs was further investigated, and results demonstrated that the mRNA expressions in DPSCs were up- and downregulated, respectively (Fig. 3A, *p* < 0.001). It was then proceeded by analyzing the cell cycle to compare distribution of DPSCs with different KAT2A expressions, and results showed that elevated KAT2A significantly decreased the ratio of G0/G1 phase and increased S phase cells; which was reversed when KAT2A was inhibited (Fig. 3B, *p* < 0.001). Moreover, the number of ALP and alizarin red stained DPSCs were raised by the overexpression of KAT2A but decreased by the inhibition of KAT2A (Fig. 3C–F, *p* < 0.01). Moreover, western blotting results revealed that COL1A1 and RUNX2 protein levels were upregulated by the elevated KAT2A and downregulated when KAT2A was inhibited (Fig. 3G).

### KAT2A promotes the succinylation of Notch1 in DPSCs

**Fig. 4:**
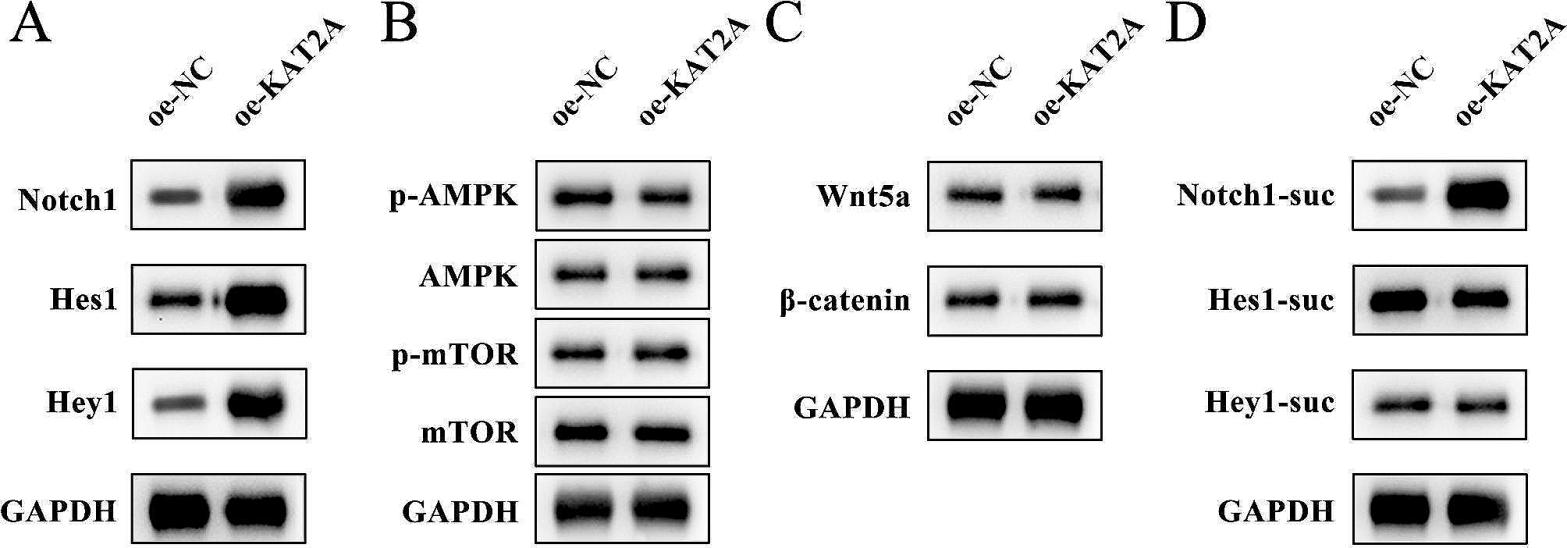
KAT2A leads to Notch1 desuccinylation. Western blotting assay was carried out to evaluate the critical protein levels of **(A)** Notch1 signaling pathway, **(B)** AMPK/mTOR signaling pathway, and **(C)** Wnt5a signaling pathway. **(D)** the Notch1 protein levels and the succinylation level of Notch1

**Fig. 5:**
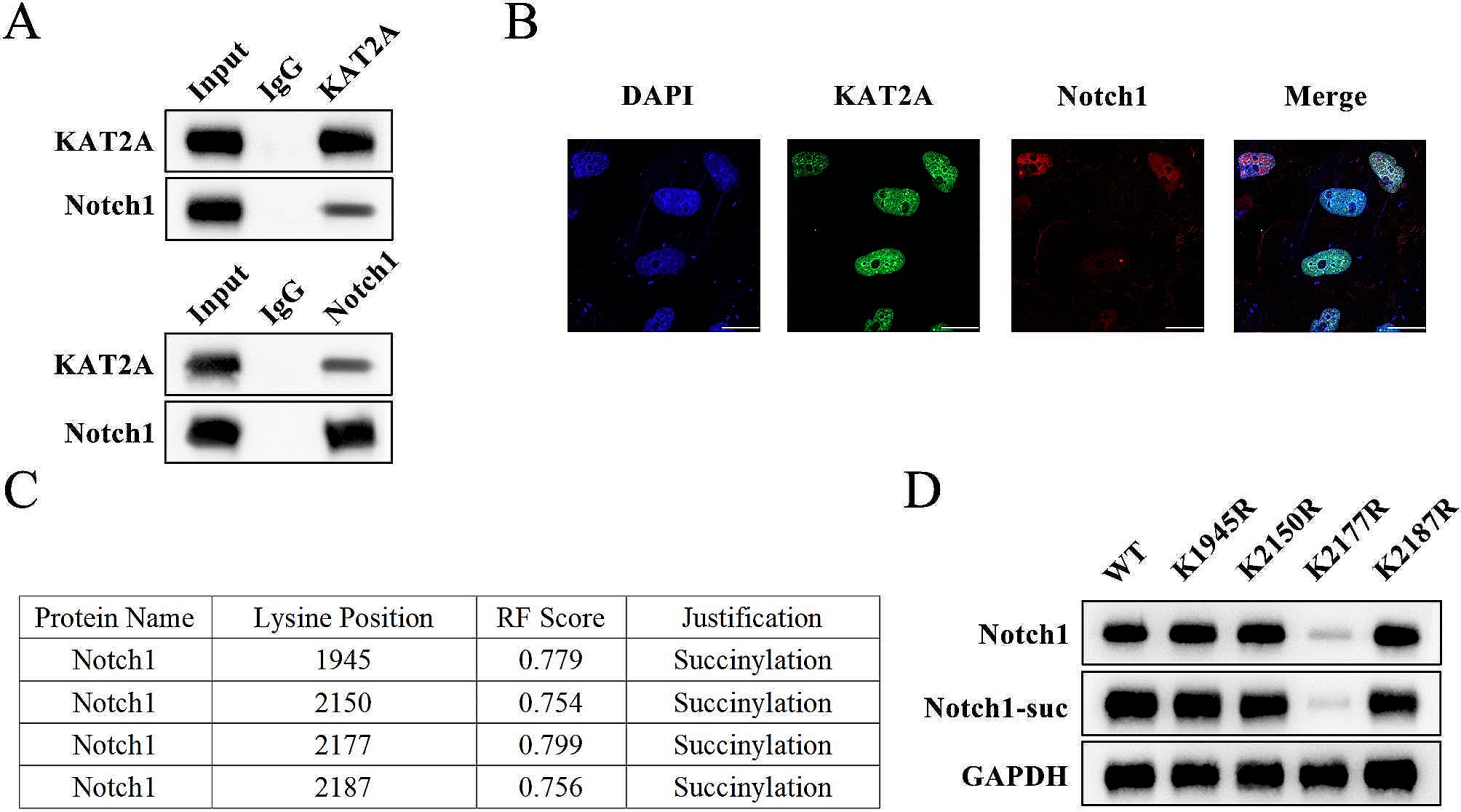
The interaction between KAT2A and Notch1. **(A)** co-IP was performed to verify the endogenous binding relationship between KAT2A and Notch1. **(B)** The co-location images of KAT2A and Notch1 accessed by the immunofluorescence method. **(C)** The predicted succinylation sites of Notch1 with DeepSuccinylSite. **(D)** Western blotting assay was carried out to assess the Notch1 protein levels and the succinylation level of Notch1 in DPSCs with overexpressed KAT2A.

Several protein levels of different signaling pathways were detected in DPSCs before and after upregulating the KAT2A expression. Results revealed that Notch1, Hes1, and Hey1 protein levels were upregulated by overexpressing KAT2A (Fig. 4A). In contrast, the expression of key proteins of AMPK/mTOR and Wnt5a signaling pathways remained independent of changes in KAT2A expression (Fig. 4B–C). The regulation of succinylation of Notch signaling by KAT2A was further investigated, and western blotting indicated overexpressed KAT2A prominently elevated the succinylated Notch1 protein levels. In contrast, the succinylated Hes1 and Hey1 protein levels were not significantly affected (Fig. 4D), suggesting KAT2A could lead to Notch1 succinylation. The interaction between KAT2A and Notch1 was then investigated via co-IP analysis, and results showed that KAT2A and Notch1 were endogenously bound to each other (Fig. 5A), where the immunofluorescence images of DPSCs demonstrated that KAT2A was bound to Notch1 in the cell nucleus (Fig. 5B). The top four sites with the highest likelihood of succinylation modification predicted by the DeepSuccinylSite were found to be K1945, K2150, K2177, and K2187 (Fig. 5C), where the four sites were mutated respectively, among which mutation of K2177 significantly decreased both the protein level and the succinylation level of Notch1 (Fig. 5D). These results collectively suggested that KAT2A succinylated Notch1 at K2177 site to increase its protein levels in DPSCs.

### Inhibition of Notch1 reversed the effects of KAT2A overexpression

**Fig. 6:**
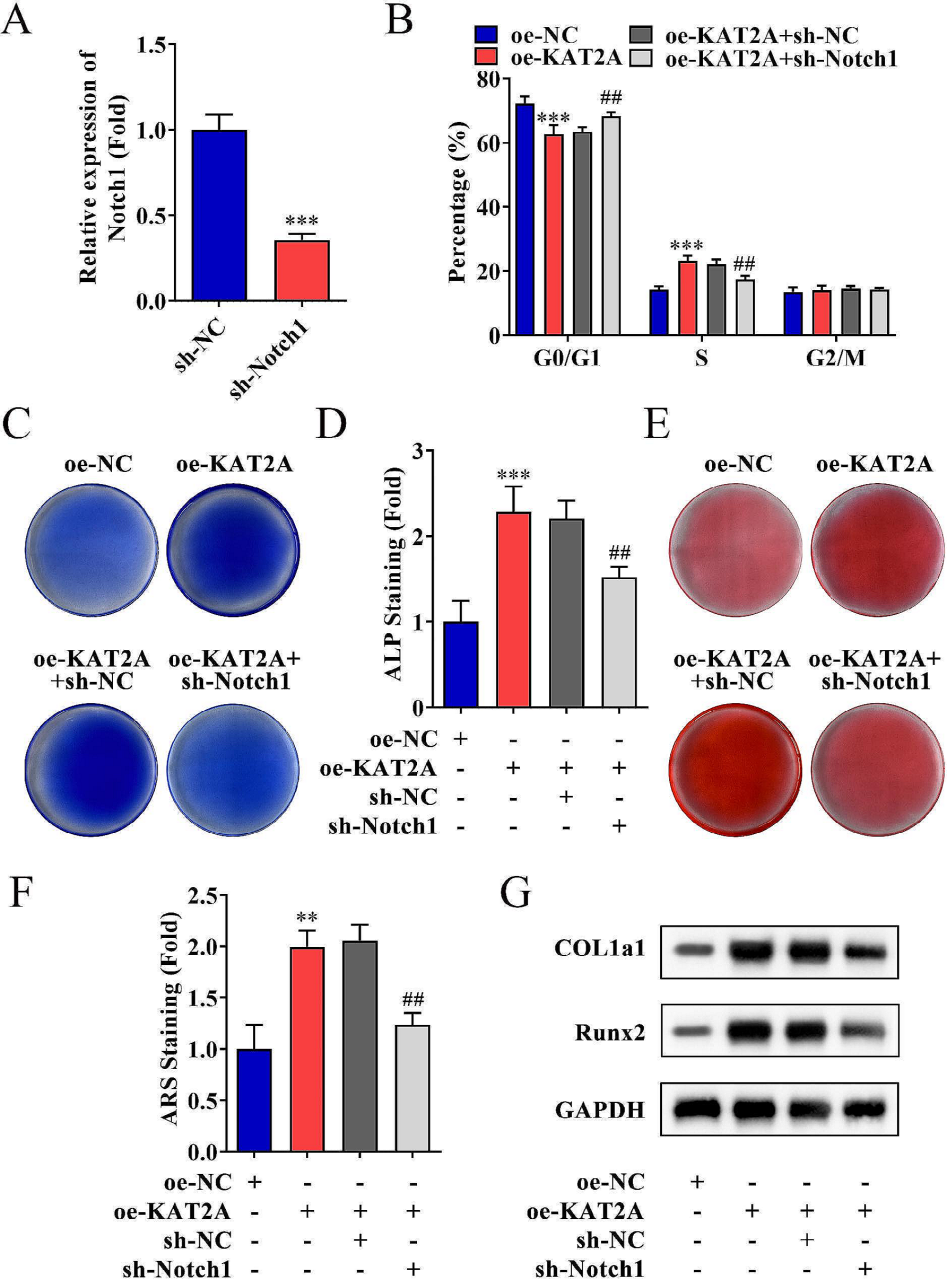
Inhibition of Notch1 reversed the effects of KAT2A overexpression. **(A)** PCR analysis was performed to evaluate the mRNA levels of Notch1 in DPSCs after transfection. **(B)** Flow cytometry detected the DPSC cell cycle phase distribution after transfection. **C–D.** Alkaline phosphatase (ALP) straining of DPSCs after seven days of osteogenic induction. **E–F.** Calcium mineralization formation was observed by alizarin red staining at day 14 in DPSCs. **G.** Protein expression of osteogenic markers COL1a1 and RUNX2 analyzed by western blotting. **p < 0.01, ***p < 0.001 (vs. oe-NC or sh-NC group); ##*p* < 0.01 (vs. oe-KAT2A + sh-NC group)

The regulatory effect of Notch1 on the proliferation and differentiation of DPSCs was investigated, and results revealed that Notch1 was successfully downregulated in DPSCs, which were also verified by PCR analysis (Fig. 6A, *p* < 0.001). The decreased ratio of G0/G1 phase and increased S phase induced by overexpressed KAT2A were significantly reversed by Notch1 inhibition (Fig. 6B, *p* < 0.01). Moreover, the mineralization state promoted by overexpressed KAT2A was inhibited by Notch1 knockdown as shown by the results of ALP and alizarin red staining (Fig. 6C–F, *p* < 0.01). Moreover, the western blotting demonstrated that COL1A1 and RUNX2 protein levels upregulated by the elevated KAT2A were downregulated by Notch1 inhibition (Fig. 6G).

## Discussion

In this study, the stem cell markers, osteogenic, adipogenic, and chondrogenic differentiation ability of DPSCs were identified, and results showed that elevated KAT2A in mineralization-inducted DPSCs promoted proliferation and differentiation. Interestingly, KAT2A increased Notch1 succinylation, which was quickly reversed by inhibiting Notch1 in regulating the DPSCs proliferation and differentiation.

DPSCs are derived from dental pulp tissue in a relatively closed pulp cavity, are not easily contaminated, and serve as excellent seed cells for tissue engineering and regenerative medicine. Compared with other osteogenic stem cells, such as tooth stem cells from deciduous teeth and dental follicle progenitor cells, DPSCs are mainly derived from the third molar and premolar teeth that need to be removed in orthodontics, with the merit of a wide donor age range and could be easily obtained. Furthermore, DPSCs, compared to bone marrow mesenchymal stem cells, show a significant ability of osteogenic differentiation [10] and can secrete some cytokines, such as brain-derived neurotrophic and hepatocyte growth factors that are lacking in bone marrow mesenchymal stem cells [12–14], which can promote the repair of systemic disease losses and have low immunogenicity, making allogeneic stem cell transplantation possible [15, 16]. In addition, a series of animal experimental studies have shown that DPSCs can regenerate nerves [17, 18], cartilage [19], and cornea [20], have a good effect on diabetes treatment [21], acute renal failure [22], cirrhosis [23], and other diseases [24]. Furthermore, DPSCs have the advantages of more accessible sampling and less trauma compared with adult stem cells distributed in blood vessels, skeletal muscle, brain bone marrow, heart, and other tissues and organs. This study isolated DPSCs from tooth pulp tissues and verified them to show differentiation capacities.

KAT2A is an acetyltransferase and succinylase that can enhance the transcriptional activity of downstream genes by regulating protein acetylation, protein succinylation, and recruiting transcriptional costimulators [10, 11]. It also induces PAX6 acetylation, promotes its ubiquitination, and its protein degradation, which is related to the proliferation and differentiation of neural stem cells [25]. Our data suggested that KAT2A was highly expressed in the DPSCs after induction of mineralization, and the high expression of KAT2A enhanced the proliferative and differentiation capacities.

Subsequently, the underlying signaling pathways that may be succinylated by KAT2A were screened. The Notch signaling pathway is highly conserved in the process of evolution, regulating cell fate through cell-cell interactions, and plays a vital role in cell proliferation, apoptosis, and differentiation [26]. The binding of Notch receptors to Delta1 and Jagged-1 ligands plays a positive and negative role in stem cell regulation, where overexpression of Notch Jagged-1 ligand activates the Notch signaling pathway or overexpression of Notch intracellular domain (NICD), inhibiting the osteogenic differentiation of DPSCs, thereby maintaining the stemness of DPSCs [27]. However, after lentiviral transfection silenced Delta1, the defect of Notch signaling inhibited the self-renewal ability of DPSCs and induced the osteogenic differentiation of DPSCs, which was detrimental to the stemness maintenance of DPSCs [28, 29]. In summary, the Notch signaling pathway plays a crucial role in DPSCs proliferation and differentiation, and its regulation is conducive to in vitro expansion and tissue regeneration of DPSCs. Our results revealed that KAT2A significantly increased the Notch1 succinylation, and the two proteins directly bound to each other at the K2177 site. Moreover, the inhibition of Notch1 partly reversed the regulatory effects of KAT2A on DPSCs. Taken together, the succinylation of Notch1 mediated by KAT2A significantly enhanced the proliferative and differentiation capacities of DPSCs. Targeting KAT2A and Notch1 to promote the proliferation and differentiation of DPSC can provide a new idea for clinical practice, such as improving the bone regeneration ability of DPSC combined with xenografts to repair craniofacial bone defects [30].

## Conclusion

In summary, KAT2A can bind with Notch1 to promote its succinylation. This mechanism leads to the maintenance of proliferative and differentiate capacities of DPSCs. Thereby, it is promising to target KAT2A and Notch1 for tooth regeneration study.

### Electronic supplementary material

Below is the link to the electronic supplementary material.

Supplementary Material 1

Supplementary Material 2

Supplementary Material 3

## Data Availability

No datasets were generated or analysed during the current study.
