# Supplementary material for: KAT2A-mediated succinylation modification of notch1 promotes the proliferation and differentiation of dental pulp stem cells by activating notch pathway
Source: BMC Oral Health. 2024 Mar 31;24:407. doi: 10.1186/s12903-024-03951-1 (PMC10981825; doi:10.1186/s12903-024-03951-1)
Supplement: Supplementary file 3 — Supplementary Material 3 [file 12903_2024_3951_MOESM3_ESM.pdf]

editage

Editing Certificate

This document certifies that the manuscript listed below has been edited to ensure language and grammar accuracy and is errorfree in these aspects. The edit was performed by professionaleditors at Editage,a brand of Cactus Communications. The author's core research ideas were not altered in any way during the editing process.The quality of the edit has beenguaranteed, with the assumption that our suggested changes have been accepted and the text has not been further altered without the knowledge of oureditors.

MANUSCRIPT TITLE

KAT2A-mediated succinylation modification of Notch1 promotes the proliferation and differentiation of dental pulp stem cells by activating Notch pathway

AUTHORS

Longwei Ye, Zeqin Yu, Lin He, Jie Yuan, Xiaodan Zhang, Lei Li, Xin Huang, Yanyan Ma, Lei Zhang

ISSUED ON

December 28, 2023

JOB CODE

BPXOO_2

Vikas Narang
Chief Operating Officer-Editage

editage helping you get published

Since 2002,Editage has helped over 430,000 authors publish around 1.2 milion research papers in scholarly journals across over 1000 disciplines through editorial,translation,transcription,and publicationsupport services.Editage is abrand of Cactus Communications (cactusglobalcom),a science communication and technology company.

CACTUS

GLOBAL:
+1(833)979-0061| request@editage.com

CHINA:
400-120-3020或021-6020-9400 |
fabiao@editage.cn

editage.com I editage.cokr I editagejp I editage.cn I editagecombr I editagecomtw I editage.de
