# Supplementary material for: KAT2A-mediated succinylation modification of notch1 promotes the proliferation and differentiation of dental pulp stem cells by activating notch pathway
Source: BMC Oral Health. 2024 Mar 31;24:407. doi: 10.1186/s12903-024-03951-1 (PMC10981825; doi:10.1186/s12903-024-03951-1)
Supplement: Supplementary file 2 — Supplementary Material 2 [file 12903_2024_3951_MOESM2_ESM.doc]

流式细胞术

F1A

| CD34 | CD45 | CD105 |
| --- | --- | --- |
|  |  |  |

油红O染色

茜素红染色

ALP染色

ALP染色

F3C

| oe-NC | oe-KAT2A | sh-NC | sh-KAT2A |
| --- | --- | --- | --- |
|  |  |  |  |

F6C

| oe-NC | oe-KAT2A | oe-KAT2A+sh-NC | oe-KAT2A+sh-Notch1 |
| --- | --- | --- | --- |
|  |  |  |  |

茜素红染色

F3E

| oe-NC | oe-KAT2A | sh-NC | sh-KAT2A |
| --- | --- | --- | --- |
|  |  |  |  |

F6E

| oe-NC | oe-KAT2A | oe-KAT2A+sh-NC | oe-KAT2A+sh-Notch1 |
| --- | --- | --- | --- |
|  |  |  |  |

WB

F3G

| COL1A1 |  |
| --- | --- |
| RUNX2 |  |
| GAPDH |  |

F4A

| NOTCH1 |  |
| --- | --- |
| HES1 |  |
| HEY1 |  |
| GAPDH |  |

F4B

| P-AMPK |  |
| --- | --- |
| AMPK |  |
| P-MTOR |  |
| MTOR |  |
| GAPDH |  |

F4C

| WNT5A |  |
| --- | --- |
| β-catenin |  |
| GAPDH |  |

F4D

| NOTCH1-SUC |  |
| --- | --- |
| HES1-SUC |  |
| HEY1-SUC |  |
| GAPDH |  |

F5A-1

| KAT2A |  |
| --- | --- |
| NOTCH1 |  |

F5A-2

| KAT2A |  |
| --- | --- |
| NOTCH1 |  |

F5D

| NOTCH1 |  |
| --- | --- |
| NOTCH1-SUC |  |
| GAPDH |  |

F6G

| COL1A1 |  |
| --- | --- |
| RUNX2 |  |
| GAPDH |  |
